# State Medical Examinations

**Published:** 1895-12

**Authors:** William Warren Potter

**Affiliations:** Buffalo, N. Y., Member New York State Medical Examining and Licensing Board


### STATE MEDICAL EXAMINATIONS.

Conducted by WILLIAM WARREN POTTER, M. D., Buffalo, N. Y.
Member New York State Medical Examining and Licensing Board.

### University of the State of New York Medical Examinations.

Examinations for license to practise medicine in this state will be held as follows :

*Dates.*—1896 : January 28–31, April 7–10, May 19–22, June 16–19.

*Places.*—New York, Albany, Syracuse, Buffalo. Each candidate is notified as to exact place.

*Daily Program.*—Tuesday, morning, 9.15—12.15, anatomy ; afternoon, 1.15—4.15, physiology and hygiene. Wednesday, morning, 9.15—12.15, chemistry ; afternoon, 1.15—4.15, surgery. Thursday, morning, 9.15—12.15, obstetrics ; afternoon, 1.15—4.15, pathology and diagnosis. Friday, morning, 9.15—12.15, therapeutics.

---

### National Confederation of State Examining and Licensing Boards.

#### REPORT OF THE COMMITTEE ON ORGANISATION.

*To the Members of the State Medical Examining and Licensing Boards.*

AT THE fourth annual session of the Conference, held at Baltimore, Md., May 9, 1895, the discussions clearly indicated a growing sentiment on the part of the medical profession throughout the country in favor of the adoption of state examinations, as affording the only effective barrier against the entrance into the profession of large numbers of ignorant and incompetent practitioners. It also became apparent that the time had fully come for the establishment of an effective central organisation to serve as the medium of communication between the several states, which could be made essentially serviceable in promoting the work of securing more nearly uniform standards of medical learning throughout the whole country.

The sentiments expressed at the meeting, concisely stated, are embodied in the following propositions :

1. That as the system of state medical licensure has been adopted in a number of states and there being a decided probability that a system of some form of state control will eventually be adopted by all the states, it is necessary that the several state examining boards should at once take measures for approximating, as nearly as possible, substantial uniformity as to ratings and standards of acquirements.

2. That there should be established at once a system of reciprocal interstate action on the part of the state examining boards, permitting the licentiate of one board to acquire a legal status under the jurisdiction of the board of another state without reëxamination, should he move from the one state to the other.

3. That measures be instituted at once for largely increasing the powers and influence of the National Conference by which it may be placed more nearly in touch with the members and representatives of state examining boards, as the most available and immediately effective means by which these desirable ends can be reached.

In order to prepare the way for carrying out these purposes a committee on organisation was appointed to draft a form of constitution or code of rules for the government of the National Conference and to outline a plan for defining its work and increasing its usefulness ; the committee to report at the next annual meeting.

The work of the National Conference is, of necessity, largely advisory and suggestive rather than mandatory ; hence its laws, while providing for an effective organisation, should be sufficiently elastic to meet the requirements of widely differing conditions. They should be framed to be helpful to all ; at the same time, if the reciprocity so greatly desired is to be accomplished through the good services of the organisation, as a species of "clearing house," there will be the need of rigidly just conditions. Hence a plan is suggested to accomplish this end, which we hope will be carefully considered by you ; we are not sure that it is best, or even feasible, to adopt this at this time. The question is, however, so important that we feel it our duty to present it to you as a suggestion.

The accompanying proposed form of a code of rules embodies, it is believed, the groundwork of an organisation of sufficient breadth and scope to meet the present demands. It is presented tentatively for inspection and criticism. And in this, the members of the committee invoke the active coöperation of the members of all the state boards of examiners, either collectively, as a board, or as individuals. They desire an expression of opinion regarding this proposed form and solicit such suggestions and criticisms as may seem pertinent and of practical utility.

It is but right to add that Dr. W. W. Potter, who was appointed on the committee, requested Dr. William Perry Watson, of Jersey City, to serve in his stead; this Dr. Watson consented to do, but as Dr. Potter was freely consulted at the request of the other members of the committee, he has also signed this report.

CONSTITUTION.

Whereas, the action in many of the states in requiring a state license before entering on the practice of medicine makes it eminently desirable that the qualifications for licensure be fairly equivalent in the various states and that, eventually, by a system of reciprocity, a physician licensed by any one board may obtain the privilege of practising under the jurisdiction of any other board by the proper certification of the license, without a new examination, a closer affiliation of the various examining and licensing boards becomes a necessity.

ARTICLE I.—NAME.

This body shall be known as the (National) Confederation of State Medical Examining and Licensing Boards.

ARTICLE II.—OBJECTS.

The Confederation has for its objects:

1. A conference of the medical examiners of the various state boards for mutual aid and counsel and for a comparison and discussion of the methods employed.

2. The collection, compilation and dissemination of information regarding state licensure in medicine and of methods for its bettering.

3. If at any time it may seem desirable, the adoption of regulations for the guidance of the operations of the confederating boards.

Or, The formulation of rulings, advisory in their nature, indicating the conclusions of the Confederation.

ARTICLE III.—MEMBERS.

SECTION 1. Any member of any examining or licensing board of any state or territory is eligible to membership while he continues a member of the board, upon presenting proper evidence of his position to the council of the Confederation, and, unless elected to membership as prescribed by this constitution, the membership will cease when he ceases to be a member of the board of examiners.

§2. Physicians, not members of an examining or licensing board, may, upon application to the council, be nominated by it to the Confederation and be elected to membership by a two-thirds vote of the members present at any meeting.

§3. The members shall have equal rights and privileges (excepting in voting for the regulations).

ARTICLE IV.—REGULATIONS.

SECTION 1. As the regulations are to be rules governing the concerted action of the confederating boards, they can be only adopted by a vote of the boards themselves.

§2. Each state shall have but one vote in voting for the regulations and the person delegated to cast the vote for any state shall be communicated by the various boards to the secretary of the Confederation, at or before any meeting at which a regulation is to be acted upon.

§3. Unless all the boards having membership in the Confederation give consent, no regulation shall be acted upon at the meeting at which it is proposed ; but it shall lie over until the next meeting and be published in the notice of the meeting.

§4. If any provision of any proposed regulation cannot be adopted by any of the confederating boards, because of the limitations of the state enactment creating the board, it must be amended before it is voted for, upon presentation of the facts by the board affected.

§5. A vote of two-thirds of the boards having membership in the Confederation is necessary for the adoption or repeal of any regulation. Or,

ARTICLE IV.—RULINGS.

SECTION 1. The Confederation shall, whenever requested by two or more boards of examiners, express an opinion on the proper procedure, standard or rating for a fair equivalence of requirements ; these shall be known as rulings.

§2. These rulings, while advisory in character, are to be expressive of the judgment of the Confederation and should be accepted by each board in affiliation with the Confederation as far as the enactments creating them will permit.

ARTICLE V.—OFFICERS.

SECTION 1. The officers of the Confederation shall be a president, who shall deliver an address at the annual meeting, two vice-presidents and a secretary, who shall act as treasurer. The officers shall be elected at the annual meeting, shall hold office for one year and until their successors are elected. They shall perform the duties usually pertaining to these positions and shall be, *ex-officio*, members of the council.

§2. There shall be elected at each meeting, at the time the officers are elected, 5 (7) of the members, who, with the officers, shall constitute the council. The council shall have charge of the affairs of the Confederation between the meetings; shall prepare and present annually to the Confederation a list of the members of the examining boards of the various states, together with the date of the termination of the service of each; shall receive and examine all applications for membership, nominating to the Confederation such, as in their opinion, should be admitted to the Confederation. It shall also order and audit all expenditures; suggest extraordinary assessments when they are deemed necessary; make preparations for the meetings; supervise the working of the regulations (rulings) and carry into effect conclusions or recommendations of the Confederation, making provision for the proper publication of all of the transactions that it is desirable to publish. All complaints or charges must be referred to the council without debate and, in like manner, any resolution or new business whatever, unless it is entertained by unanimous consent. All business referred to it must be reported back to the Confederation at the same meeting, unless it is specifically instructed to the contrary. It shall make rules for its own government and make an annual report to the Confederation.

ARTICLE VI.—MEETINGS.

The confederation shall hold one meeting annually at such time and place as the council may select, but additional meetings for specific purposes may be called by the council if it shall be deemed necessary; at least two weeks' notice must be given of any special meeting and the business to be transacted specified in the call for the meeting.

ARTICLE VII.—FUNDS.

SECTION 1. Each state board represented in the Confederation shall pay an annual fee of $10 into the treasury and shall be debarred from the privileges of the Confederation when in arrears. In consideration of this fee, the members of the boards so contributing shall be exempt from the payment of the regular dues.

§2. Except as provided in Section 1, the members of the Confederation shall pay an annual fee of $1.00 into the treasury and no one shall have the privilege of registering at any meeting until all arrearages are paid.

§3. If at any time it may be necessary, the Confederation, upon recommendation of the council, may impose an extra assessment, which shall be levied on all members alike.

ARTICLE VIII.—QUORUM.

Five members shall be a quorum to transact business, except on a vote on a regulation, but the board of examiners of any state may transmit to the secretary of the Confederation its vote on any regulation ; this vote shall be received the same as if cast by a member of the board present at the meeting.

ARTICLE IX.—AMENDMENTS.

Amendments to the constitution must be submitted in writing to the council at least thirty days before the meeting, be advertised in a notice of the meeting and adopted by a two-thirds vote of the members present ; provided, any state board of examiners shall have the privilege of transmitting its vote to the secretary of the Confederation, which shall be received the same as if it were cast by a member present at the meeting ; but this privilege is not accorded to any board represented by any of its members at the meeting.

THE COMMITTEE OF ORGANISATION.

---

NEW MEXICO TERRITORIAL BOARD OF MEDICAL EXAMINERS.

IN THE territory of New Mexico the territorial board of health exercises the functions of the board of medical examiners.

In 1882, there was adopted by the legislature of New Mexico, and approved, a statute regulating the practice of medicine, its details resembling the then existing statute of Illinois. But, being unsatisfactory to the medical profession and adversely criticised by the bar, various futile efforts were made by the New Mexico Medical Society to secure a better statute. In September, 1894, the territorial board of medical examiners, in session at Albuquerque, appointed a committee of three of its members, who, with the president of the board and the president of the New Mexico Medical Society, should prepare a bill looking to a better statute. In January, 1895, such a bill was ready and, having met the approval of a majority of the board, and of the officers of the New Mexico Medical Society, was presented to the legislature then in session. It became a law, but in its passage received several unnecessary alterations. An attempt was made in drafting this bill to utilise the best materials from kindred statutes throughout the United States, as well as our own considerable experience.

The following-named physicians comprise the membership of the board for 1895: W. R. Tipton, M. D., president, Las Vegas; G. S. Easterday, M. D., vice-president, Albuquerque; Francis H. Atkins, M. D., secretary, East Las Vegas; J. H. Sloan, M. D., Treasurer, Santa Fe; Wm. Eggert, M. D., Santa Fe; J. J. Shuler, M. D., Raton; J. M. Cunningham, M. D., East Las Vegas.

---

### THE OREGON MEDICAL PRACTICE LAW.

THE legislative assembly of the State of Oregon, in its 1895 session, passed a new law for the regulation of the practice of medicine and surgery, which, in some of its features, is of more than local interest. Among other things, it provides that the Governor shall appoint five persons from among the most competent physicians of the state, all of whom shall have been residents of the state for seven years and of at least five years' practical experience in their profession, who shall be known as the Board of Examiners for the State of Oregon. Three of the board shall be regulars, one "eclectic" and one "homeopathist." The full term of office is five years. The board is required to hold meetings for examination on the first Tuesday of January and July of each year, at Portland, and it may call special meetings when in the opinion of a majority of the members it is deemed necessary.

Examinations shall be in anatomy, physiology, chemistry, materia medica, therapeutics, practice of medicine, surgery, obstetrics, diseases of women, medical jurisprudence and such other branches as the board shall deem advisable. They must be both scientific and practical, and of sufficient severity to test the candidates' fitness to practise medicine and surgery. They may be written or printed, or partly printed and partly printed, questions and answers.

The board may refuse or revoke a license for unprofessional or dishonorable conduct, subject, however, to the right of the applicant to appeal from the decision of the board to the circuit court. And before a license can be revoked, a complaint of some person under oath must be filed in the office of the secretary of the board, charging the acts complained of ; the accused must be served with a written notice and copy of such complaint ; a time and place for hearing thereon appointed, and stated in the notice, and the accused be given an opportunity to appear and defend himself, personally and by counsel, have the sworn testimony of witnesses taken and present other evidence in his behalf.

The words "unprofessional" or "dishonorable conduct," are declared to mean (1) the procuring or abetting in procuring a criminal abortion ; (2) the employing of what are popularly known as "cappers" or "steerers ;" (3) the obtaining of any fee on the assurance that a manifestly incurable disease can be permanently cured ; (4) the wilfully betraying of a professional secret ; (5) all advertising of medical business in which untruthful and improbable statements are made ; (6) all advertising of any medicines, or of any means whereby the monthly periods of women can be regulated, or the menses reëstablished if suppressed ; (7) conviction of any offense involving moral turpitude ; (8) habitual intemperance.

The person receiving a license shall file the same, or a copy thereof, with the county clerk of the county wherein he resides, and in case he shall move into another county, he shall procure from the county clerk a certified copy of such license and file the same with the county clerk in the county to which he removes.

The penalty for practising medicine or surgery without a license, or contrary to the law, is a fine of not less than $50 or more than $100, or imprisonment in the county jail not less than ten nor more than ninety days, or both such fine and imprisonment. All fines are to be paid into the state treasury for the use and benefit of the common schools.

Any person shall be regarded as practising medicine within the meaning of this act who shall append the letters "M. D." or "M. B." to his or her name, or, for a fee, prescribe, direct or recommend for the use of any person, any drug or medicine or agency for the treatment, care or relief of any wound, fracture, or bodily injury, infirmity or disease ; provided, however, the act shall not apply to dentists in the practice of their dental profession. —*Jour. Am. Med. Assn.*

